# Does the cytokine adsorber CytoSorb^®^ reduce vancomycin exposure in critically ill patients with sepsis or septic shock? a prospective observational study

**DOI:** 10.1186/s13613-022-01017-5

**Published:** 2022-05-23

**Authors:** Christina Scharf, Ferdinand Weinelt, Ines Schroeder, Michael Paal, Michael Weigand, Michael Zoller, Michael Irlbeck, Charlotte Kloft, Josef Briegel, Uwe Liebchen

**Affiliations:** 1 Department of Anesthesiology, University Hospital, LMU Munich, Marchioninistr. 15, 81377 Munich, Germany; 2 Department of Clinical Pharmacy and Biochemistry, Institute of Pharmacy, Freie Universität Berlin, Kelchstr. 31, 12169 Berlin, Germany; 3 Graduate Research Training Program PharMetrX, Freie Universität Berlin/Universität Potsdam, Berlin, Germany; 4 Institute of Laboratory Medicine, University Hospital, LMU Munich, Marchioninistr. 15, 81377 Munich, Germany

**Keywords:** Vancomycin, Critically ill patients, CytoSorb^®^, Sepsis, Pharmacokinetics, Adsorption

## Abstract

**Background:**

Hemadsorption of cytokines is used in critically ill patients with sepsis or septic shock. Concerns have been raised that the cytokine adsorber CytoSorb^®^ unintentionally adsorbs vancomycin. This study aimed to quantify vancomycin elimination by CytoSorb^®^.

**Methods:**

Critically ill patients with sepsis or septic shock receiving continuous renal replacement therapy and CytoSorb^®^ treatment during a prospective observational study were included in the analysis. Vancomycin pharmacokinetics was characterized using population pharmacokinetic modeling. Adsorption of vancomycin by the CytoSorb^®^ was investigated as linear or saturable process. The final model was used to derive dosing recommendations based on stochastic simulations.

**Results:**

20 CytoSorb^®^ treatments in 7 patients (160 serum samples/24 during CytoSorb^®^-treatment, all continuous infusion) were included in the study. A classical one-compartment model, including effluent flow rate of the continuous hemodialysis as linear covariate on clearance, best described the measured concentrations (without CytoSorb^®^). Significant adsorption with a linear decrease during CytoSorb^®^ treatment was identified (p < 0.0001) and revealed a maximum increase in vancomycin clearance of 291% (initially after CytoSorb^®^ installation) and a maximum adsorption capacity of 572 mg. For a representative patient of our cohort a reduction of the area under the curve (AUC) by 93 mg/L*24 h during CytoSorb^®^ treatment was observed. The additional administration of 500 mg vancomycin over 2 h during CytoSorb^®^ attenuated the effect and revealed a negligible reduction of the AUC by 4 mg/L*24 h.

**Conclusion:**

We recommend the infusion of 500 mg vancomycin over 2 h during CytoSorb^®^ treatment to avoid subtherapeutic concentrations.

*Trial registration* NCT03985605. Registered 14 June 2019, https://clinicaltrials.gov/ct2/show/NCT03985605

## Introduction

Sepsis and septic shock are defined as life-threatening organ dysfunction caused by dysregulated host response to severe infections [1, 2]. Reported prevalence rates of sepsis range from 12% (USA) to 27% (UK) of all critically ill patients [3]. Inflammatory cytokines play a pivotal role in the progression of sepsis and cause a dysregulation of vital organ functions possibly leading to organ failure and death [2, 4]. Treatment of sepsis and septic shock includes (among other measures) timely, effective antibiotic therapy, which is in most cases the only causal therapeutic option [1, 5, 6]. Vancomycin is a glycopeptide antibiotic that is used for combination therapy in sepsis due to its broad spectrum against Gram-positive pathogens including methicillin-resistant *S. aureus* [7]. Antimicrobial activity of vancomycin is linked to the ratio of the area under the concentration time curve (AUC) to the minimal inhibitory concentration (MIC) being ≥ 400 mg/L*24 h [8]. Due to known pharmacokinetic (PK) alterations in critically ill patients (i.e., augmented renal clearance, increased volume of distribution) and nephrotoxic side effects at higher plasma concentrations, therapeutic drug monitoring (TDM) combined with continuous infusion is recommended for vancomycin in serious infections [9, 10].

A more recent therapeutic option is hemadsorption of cytokines to restore "immune homeostasis" in the treatment of the dysregulated inflammatory state of septic shock [4]. CytoSorb^®^ (CytoSorbents Corporation, NJ, USA) is licensed for extracorporeal cytokine elimination in hyperinflammatory conditions within the European Union since 2011 [11]. To date, the CytoSorb^®^ has already been used 121.000 times worldwide [12]. The cartridges can be easily installed within ordinary hemodialysis-, hemofiltration-, extracorporeal membrane oxygenation- and heart–lung-machines. The mode of action is based on the adsorption of cytokines by highly porous high-tech polymer beads with a large surface area of about 45,000 m^2^. Molecules up to a molar mass of 55 kDa can potentially be adsorbed by the filter (molar mass range of cytokines 6–70 kDa, molar mass of vancomycin 1.45 kDa) due to hydrophobic interactions and therefore eliminated from the patient [12, 13].

In addition to its utility in sepsis therapy, CytoSorb^®^ is also used to rapidly eliminate drugs in case of intoxications [14–16]. The ability of the CytoSorb^®^ filter to adsorb drugs suggests that this might also happen unintentionally. Indeed, previously published in vitro data indicated significant adsorption of antibiotics by CytoSorb^®^ [17, 18]. Of particular interest is the interaction between CytoSorb^®^ and vancomycin. However, there is a lack of reliable clinical data supporting the adsorption of vancomycin by CytoSorb® [19, 20]. As a consequence, intensified TDM of antibiotics was recommended during the use of CytoSorb^®^, although this service is sometimes not available, especially during weekends [4, 11, 17, 21, 22].

The aim of this prospective observational study was to quantify the adsorption of vancomycin by CytoSorb^®^. For this purpose, TDM data in critically ill patients during and without CytoSorb^®^ treatment were analyzed. A population PK model approach including adsorption models was used to investigate the influence of the CytoSorb^®^ adsorber on vancomycin serum concentrations and to give a recommendation on vancomycin dose adjustment.

## Material and methods

### Data and patients

Critically ill patients from a prospective observational study (Trial registration NCT03985605. Registered 14 June 2019, https://clinicaltrials.gov/ct2/show/NCT03985605) on continuous venovenous hemodialysis (CVVHD) or hemodiafiltration (CVVHDF) with at least two serum vancomycin samples each during and without CytoSorb^®^ therapy were included. The study protocol was approved by the Institutional Review Board of the Medical Faculty of the LMU Munich (registration number 18–578). NCT03985605 is a monocentric study at the tertiary care University Hospital Munich, LMU. In this study, patients with anti-infective therapy and routine TDM measurements have been prospectively observed since 2018. An interim analysis was carried out in May 2021 to analyze how many patients were on CytoSorb^®^, CVVHD(F) and vancomycin therapy (this kind of subgroup analysis was already planned at the beginning of the study). All patients fulfilling the specific inclusion criteria were used to address the present research question. Vancomycin dosing regimens were administered according to the assessment of the responsible physician. Total serum concentrations were quantified with the online TDM Vancomycin 3rd Gen immunoassay on a Cobas^®^ 8000 c702 modular analyser (Roche Diagnostics, Mannheim, Germany). According to recent recommendations for intensive care patients, steady-state concentrations between 20 and 25 mg/L were defined as the therapeutic target range and are linked to an effective but non-toxic AUC [23]. CytoSorb^®^ and renal replacement therapy were initiated and controlled by the responsible physician, independent of this study. Demographic patient data (sex, age, weight) and laboratory data (serum albumin concentration, serum creatinine concentration) were collected.

### Population pharmacokinetic modeling/model development

Since all patients received continuous vancomycin infusions, only a one compartmental disposition model was investigated. Interindividual variability (IIV) was implemented in a stepwise process using exponential models. Different residual variability models (additive, proportional and combined) were considered. Models were evaluated and discriminated based on the objective function value (OFV), precision of the PK parameter estimates and goodness-of-fit plots. Patient and dialysis characteristics—other than CytoSorb^®^-treatment—potentially influencing vancomycin PK were investigated as possible covariates. Candidates for the covariate analysis were pre-selected based on graphical exploration and literature information. In addition to statistical significance (alpha-error level < 0.05, i.e., ∆OFV < −3.84 for the integration of one additional parameter), covariate selection was based on the reduction in unexplained variability, higher precision of parameter estimates, biological plausibility and clinical relevance. Log-likelihood profiling was performed to determine confidence intervals (CI) of population parameters.

Modelling activities were performed in NONMEM 7.4 (ICON Development and Solutions, Ellicott City, MD, USA), PsN 4.7.0 [24] and Pirana 2.9.9 [25] using the first-order conditional estimation with interaction method. Graphical and statistical analysis, and simulations (mrgsolve package) were performed in R/Rstudio (R version 4.02, CRAN.R-project.org).

### Effect of CytoSorb®-treatment on vancomycin concentrations

Two distinct approaches were chosen to investigate the effect of CytoSorb^®^-treatment on vancomycin concentrations and hence PK parameters. Based on established definitions and previous work on the topic a maximal increase of vancomycin clearance > 10% during CytoSorb^®^ therapy was defined as mild adsorption, an increase > 100% as moderate and > 400% as strong adsorption [26, 27]:

1. *CytoSorb*^*®*^*-treatment as categorical covariate on clearance*
  The potential effect of CytoSorb^®^-treatment on vancomycin clearance was investigated as categorical covariate (i.e., CytoSorb^®^ on or off, i.e., constant adsorption effect over time) with a proportional clearance increase during CytoSorb^®^-treatment. A statistically significant drop in objective function value (∆ OFV < −3.84) and a precise and plausible estimate for the parameter characterizing the covariate effect/extent of clearance increase would indicate an adsorption of vancomycin by the CytoSorb^®^ filter.
2. *Saturable adsorption models*

Two adsorption models of vancomycin as decreasing adsorption effect over time during CytoSorb^®^ treatment were examined: a linear or a hyperbolic decrease in adsorption rate constant. In the linear (Eq. 1) and the hyperbolic decrease models (Eq. 2) the adsorption rate is linked to the maximum adsorption rate (${k}_{\text{max}}$), the drug amount already adsorbed at the filter (${A}_{\text{Cytosorb}}(t)$) and either the maximum drug amount that can be adsorbed (${A}_{\text{max}}$) or the drug amount associated with half of the maximum adsorption capacity (${A}_{50}$):

1 $${CL}_{\text{Cytosorb}}\left(t\right)= {V}_{1} \cdot {k}_{\text{max}}\cdot \left(1-\frac{{\mathrm{A}}_{\text{Cytosorb}}\left(t\right)}{{A}_{\text{max}}}\right).$$

2 $${CL}_{\text{Cytosorb }}\left(t\right)= {V}_{1} \cdot \frac{{k}_{\text{max}}*{A}_{50}}{{A}_{\text{Cytosorb}}\left(t\right)+ {A}_{50}}.$$

A statistically significant drop in objective function value and precise and plausible parameter estimates would indicate an adsorption of vancomycin by the CytoSorb^®^ filter.

### Recommendations for vancomycin dosing adaptions

Based on the structural PK parameter estimates and interindividual variability of the final model, stochastic simulations (*n* = 3000) were performed to assess vancomycin exposure, i.e., AUC, with and without CytoSorb^®^. In particular, the simulations examined how much the vancomycin exposure is reduced by the CytoSorb^®^ to assess the clinical relevance and investigate necessary dose adaptations. The AUC was employed as the relevant PK/PD index and calculated for the 24 h during CytoSorb^®^ installation. In a first step, the lowest maintenance infusion rate exceeding a median vancomycin steady state concentration of 20 mg/L without CytoSorb^®^ was determined. In a second step, the influence of the CytoSorb^®^ on the exposure was assessed in steady state.

## Results

### Data and patients

**Fig. 1:**
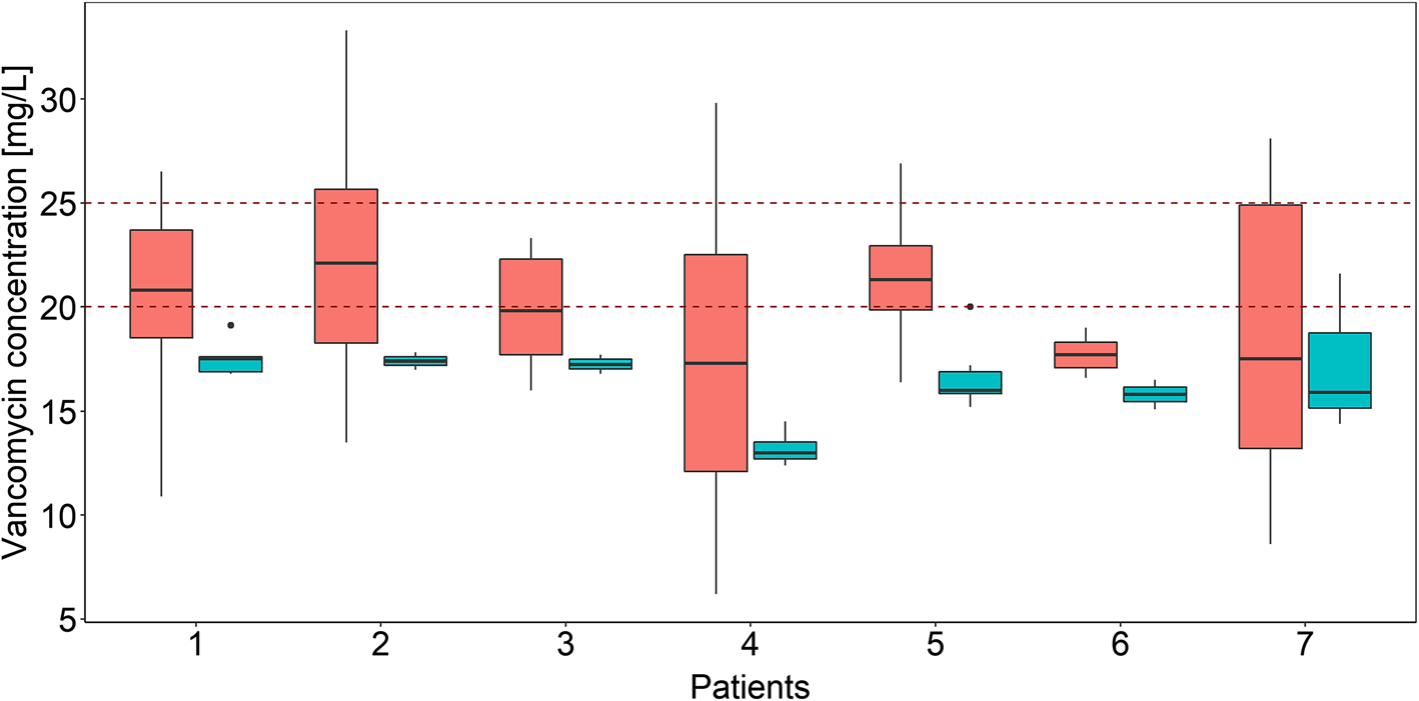
Boxplot of vancomycin concentration during (turquoise) and without (red) CytoSorb® therapy across patients. Lower and upper box boundaries 25th and 75th percentiles, respectively; line inside box: median; lower and upper error lines: 10th and 90th percentiles, respectively; points: data falling outside 10th and 90th percentiles; dashed red line: target concentration for vancomycin

A total of 160 vancomycin serum samples from 7 patients with septic shock were included in the analysis (see Table 1). 15% of the samples (*n* = 24) were collected during CytoSorb^®^-treatment. The cohort of patients studied was relatively young (20–52 years), severely ill (median SOFA score on study day 1: 17, range 15–20) and had a low residual diuresis (median 0 mL/day, range 0–3550 mL/day). All patients received vancomycin as a continuous infusion with a preceding loading dose over 2 h (median loading dose: 1500 mg. range: 250–2000). The median infusion rate was 58 mg/h (range: 20–125 mg/h). Vancomycin concentrations during CytoSorb^®^ therapy were found to be significantly lower than without CytoSorb^*®*^ therapy (median concentration during vs. without CytoSorb^®^: 16.7 vs. 20.4 mg/L, p< 0.001, *t*-test), although the infusion rate was significantly higher during CytoSorb^®^ (median infusion rate during vs. without CytoSorb^®^: 70 vs. 40 mg/h, p< 0.001, *t*-test). Figure 1 illustrates that the measured concentrations were decreased in all patients during CytoSorb^®^ therapy. Of the 24 concentration measurements during CytoSorb^®^ therapy, only two attained the target concentration of 20 mg/L, i.e., 95.6% of these measurements were subtherapeutic. However, 48.9% of the concentrations without CytoSorb^®^ were in the subtherapeutic range as well.

**Table 1:** Patient (at baseline), treatment and blood sampling characteristics

Patient characteristic	Total dataset
Categorical variables	n (%)
No. of patients	7 (100)
No. of male patients	6 (86)
No. of patients with CVVHD/CVVHDF	7 (100)/ 3 (43)^a^
No. of patients with Cytosorb®	7 (100)
No. of samples	160 (100)
No. of CytoSorb®-treatments	20
No. of samples during Cytosorb®	24 (15)
Continuous parameters [unit]	Median (range)
CytoSorb®-treatment duration [h]	6 (1.7–27.9)
Vancomycin daily dose [mg]	1380 (240–3000)
Vancomycin concentration with CytoSorb® [mg/L]	16.7 (12.4–21.6)
Vancomycin concentration without CytoSorb® [mg/L]	20.4 (6.2–33.3)
Continuous parameters on study day 1 [unit]	Median (range)
Age [years]	52 (20–57)
Weight [kg]	87 (56–130)
Serum albumin concentration [g/dL]	2.4 (1.1–4.2)
SOFA at day 1	17 (15–20)
Bilirubin concentration [mg/dL]	6.1 (0.1–31.4)
IL-6 concentration [pg/mL]	102 (4.5–554,000)
CRP concentration [mg/dL]	7.5 (0.1–49.4)
Residual diuresis [mL/day]	0 (0–3550)
Dialysate flow [L/h]	2.0 (1.5–3.0)
Substitute flow [L/h]^b^	1.5 (1.5–3.0)
Blood flow [L/h]	6.0 (4.8–12)

*SOFA* Sequential Organ Failure Assessment [28], *CRP* C-reactive protein, *CRRT* continuous renal replacement therapy, *CVVHD* continuous venovenous hemodialysis, *CVVHDF* continuous venovenous hemodiafiltration, IL-6 interleukin 6

^a^In 3 patients the dialysis-type was switched

^b^When *CVVHDF* on

### Population pharmacokinetic modeling

**Fig. 2:**
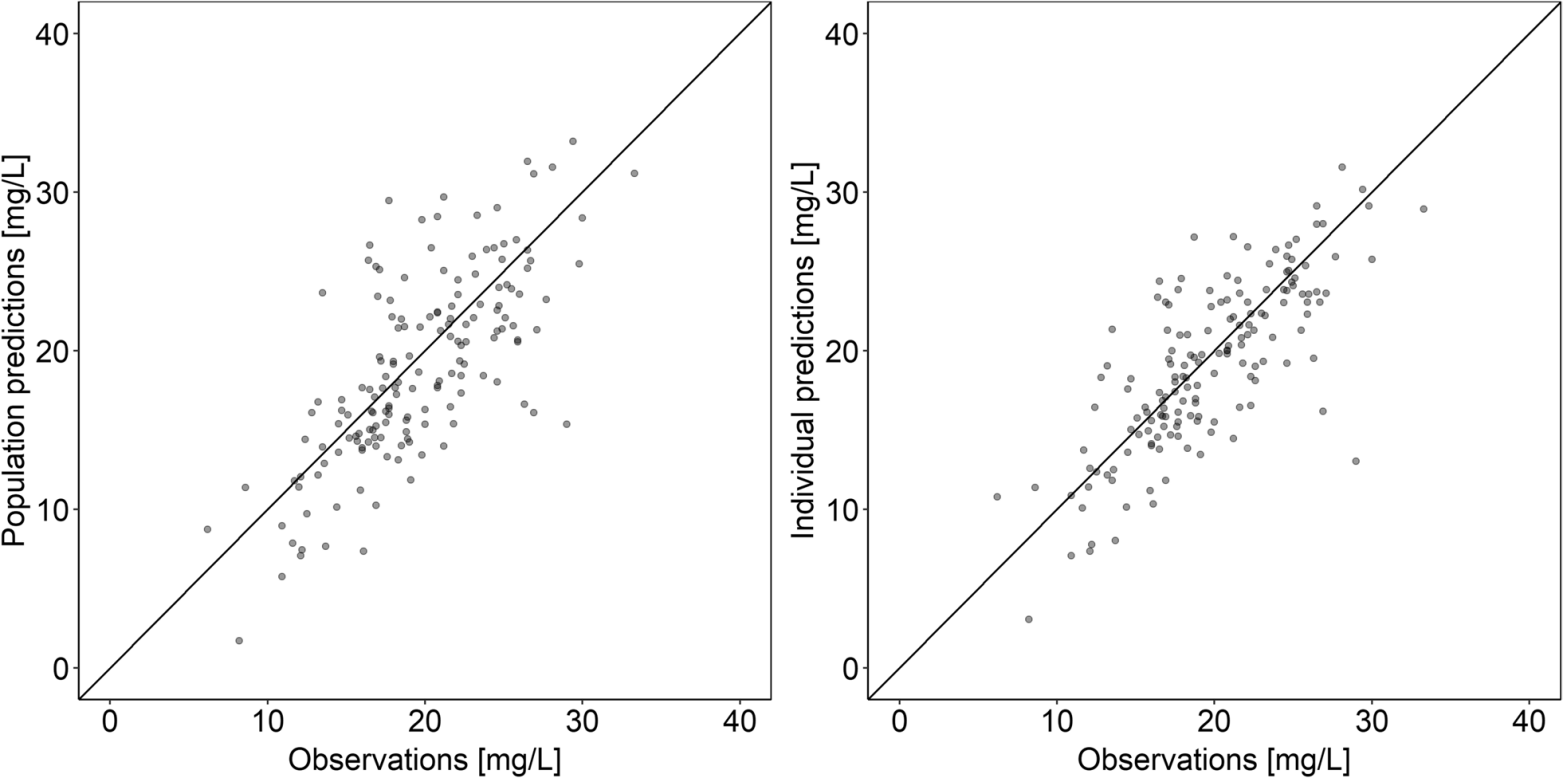
Goodness-of-fit plots for the final population pharmacokinetic model of vancomycin in critically ill patients undergoing renal replacement therapy and CytoSorb® therapy. Points: observations, lines: line of unity

A one-compartment PK disposition model with zero-order input and first-order elimination, IIV on clearance (CL) and an additive residual variability model best described the data. In a first step, the effluent flow rate implemented as a linear covariate on CL significantly improved the fit and was therefore included in the model (*p*-value < 0.001, ∆ OFV: -16.0). This covariate effect translates into an increase of the total CL by 60% when the effluent flow rate is doubled from 2.0 to 4.0 L/h. Implementing the adsorption model describing a linear decrease of the CL (Eq. 1) revealed the best model fit and led to another significant drop in OFV (∆ OFV: -32.9, see Fig 2). Due to the limited number of available samples and to avoid overparameterization, volume of distribution and the covariate effect of the effluent flow rate on CL had to be fixed in the final model to previously estimated values. A maximum adsorption capacity of 572 mg (90% CI: 305–1750 mg) and a maximum increase of total vancomycin clearance by 291% (90% CI: 147%–522%) immediately after installation of the CytoSorb^®^ (maximum CL with vs without CytoSorb^®^: 8.96 vs. 2.29 L/h) was estimated. The final model parameters are displayed in Table 2.

**Table 2:** Parameter estimates for the final pharmacokinetic model

	Parameter estimates (90% confidence interval) [shrinkage %]
Parameter [unit]	
Fixed-effect parameters	
CL [L/h]	2.29 (1.97–2.64)
V [L]	98.1^a^
COV_Effluent flow rate_ on CL	0.0003^a^
*k*_max_ [h^−1^]	0.068 (0.0345–0.122)
*A*_max_ [mg]	572 (305–1750)
Interindividual variability	
CL, CV %	14.4 (0.8–29.9) [6]
Residual variability	
Additive error [mg/L]	3.55 (3.18–3.98) [2]

*CL* clearance, *V* volume of distribution, *CO*V covariate, *k*_max_ maximum adsorption rate constant, *A*_max_ maximum drug amount adsorbed at the CytoSorb® filter, *CV* coefficient of variation

^a^Parameter fixed to previously estimated parameters. Individual CL = CL_typical value_ * (1 + COV_effluent flow rate_* (effluent flow rate—2000)) + V * *k*_max_ * (1−A_Cytosorb_(t)/*A*_max_)

### Recommendations for vancomycin dosing adaptions

**Fig. 3:**
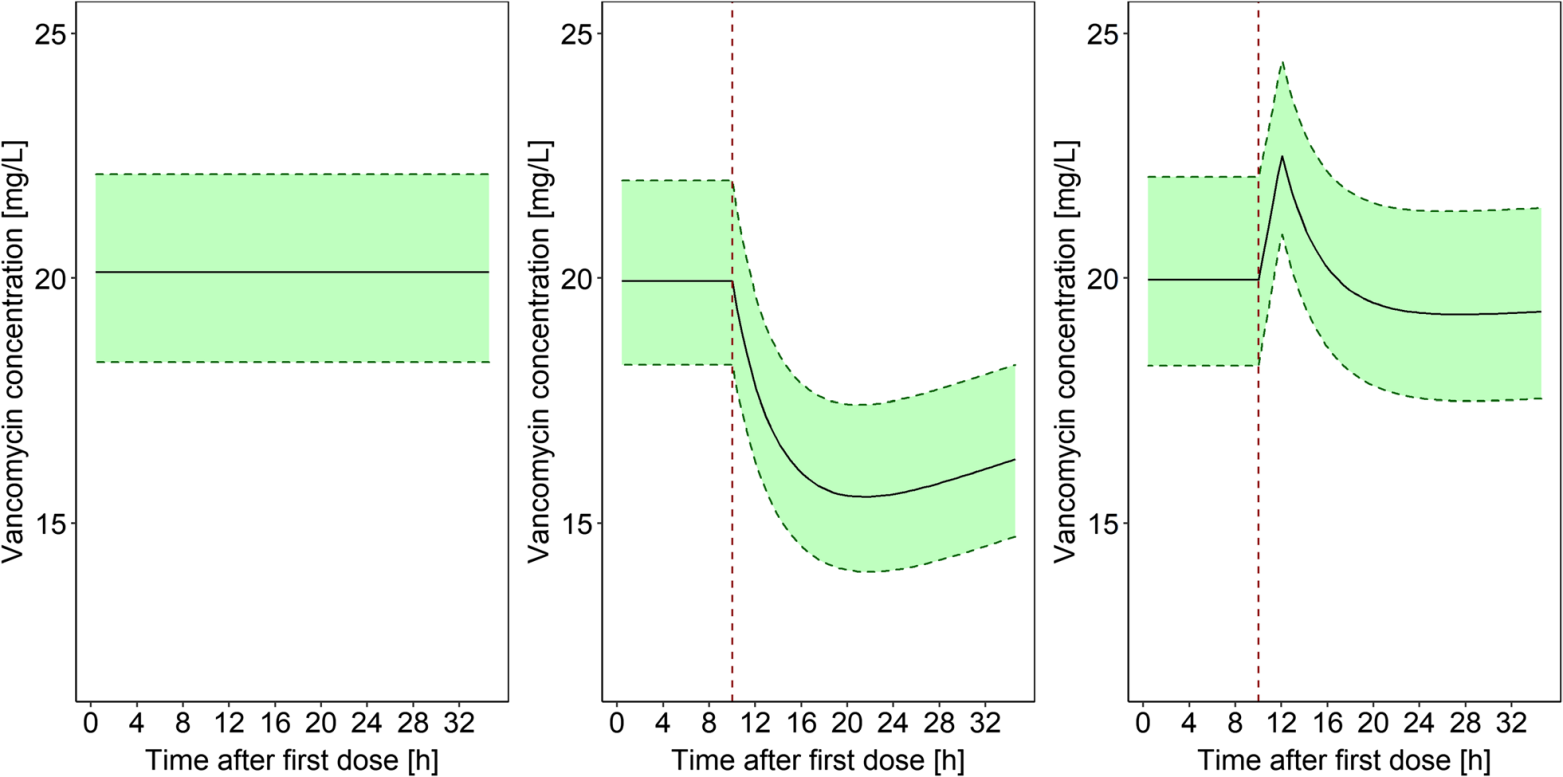
Predicted (n = 3000) vancomycin pharmacokinetic plasma profile during continuous infusion. Left: without CytoSorb®. Middle: with CytoSorb® installation after 10 h and no additional vancomycin dose. Right: with CytoSorb® installation after 10 h and an additional vancomycin dose of 500 mg over 2 h. For all scenarios, a continuous infusion of 46 mg/h was administered. Black line: median prediction, Green shade: 50% prediction interval, red vertical line: insertion of the CytoSorb®

The final model (including the adsorption submodel with linear decrease of the adsorption rate) was used to investigate the clinical relevance of the CytoSorb^®^ on vancomycin exposure and to derive a dosing recommendation. All simulations were performed for a patient with the median dialysis intensity of our population (effluent flow rate=2000 mL/min). For this representative patient, a vancomycin infusion rate of 46 mg/h resulted in a median steady-state plasma concentration of slightly above 20 mg/L without CytoSorb^®^ therapy (see Fig. 3). Immediately after the installation of a CytoSorb^®^, the plasma concentration dropped significantly to ~ 16 mg/L and increased again only slowly thereafter remaining persistently in the range of 16–20 mg/h during the next 24 h. The median AUC in the first 24 h after CytoSorb^®^ installation was reduced by 93 mg/L*24 h (median AUC: 481 without vs. 388 mg/L*24 h with CytoSorb^®^). If an additional dose of 500 mg was administered over 2 h at the time of CytoSorb^®^ installation, the effect of the CytoSorb^®^ was largely compensated with a median AUC of 477 mg/L*24 h (see Fig. 3).

## Discussion

Our study revealed a significant adsorption of vancomycin by the CytoSorb^®^ device. To achieve therapeutic exposure, additional dosing is mandatory. Even the mere observation of the measured vancomycin concentrations during CytoSorb^®^ demonstrated how challenging the dosing of vancomycin during CytoSorb^®^ therapy is (22 of 24 samples subtherapeutic). The responsible physicians dosed significantly higher during CytoSorb^®^ therapy, and one might speculate that they tried to compensate for the loss by adsorption during CytoSorb^®^ therapy (if adsorption was suspected). However, the mere observation of concentrations could be misleading, as no steady-state conditions were met, and dialysis intensity is not taken into account. A better way, as this work shows, is to use population PK approaches to quantitatively investigate a heterogeneous, real-world clinical dataset with time-varying variables. The effluent flow rate proved to be an influencing covariate in the graphical and statistical analysis which demonstrates that this covariate is relevant when considering the measured concentrations. This result is in agreement with previous studies that revealed an effect of dialysis intensity on drug concentrations in general [29–31] and specifically for vancomycin [32].

To the best of our knowledge, no previous quantitative clinical data and limited overall data on vancomycin adsorption by CytoSorb^®^ is available and our study is therefore the first to provide quantitative insights in vivo. Reiter et al. presented the first in vitro data on the adsorption of vancomycin on a predecessor product of CytoSorb^®^ (Betasorb^®^) in uremic blood. Their study showed almost complete adsorption of vancomycin within the first hour after the start of the experiment, although no therapeutic concentrations were considered (1048 mg/L) in the experimental set-up [18]. König et al. confirmed these results years later in human albumin solution for the CytoSorb^®^ investigating therapeutic concentrations as a baseline [17]. Besides the in vitro data, there is only limited clinical data: Dimski et al. demonstrated a significant adsorption and a superiority of the continuous infusion in two patients with vancomycin therapy and simultaneous CytoSorb^®^ application [19]. Scandroglio et al. retrospectively showed an increased daily vancomycin dose requirement of about 500 mg in 89 critically ill patients [20]. Unfortunately, no population pharmacokinetic approach was applied in either study, so that a quantitative evaluation and comparability to our study is not possible without restrictions. In addition, no specific dosing recommendations could be derived from these studies. However, it should be noted, that the increased daily dose requirement of about 500 mg supports the results of our study.

Considering that recommended daily doses of vancomycin in critically ill patients on continuous renal replacement therapy (without CytoSorb^®^ treatment) vary between 500 and 1500 mg (median in the present study: 1380 mg), it seems crucial to compensate for a loss of 572 mg [33–36]. The observed reduction of the AUC by 93 mg/L*24 h (relative reduction: 19%) confirms this. The simulations carried out provide a clinically feasible option of circumventing the problem of vancomycin adsorption by the CytoSorb^®^. It should be emphasized that this additional dose should be administered each time the CytoSorb^®^ is replaced.

The previous recommendation to perform intensified TDM of antibiotics when using CytoSorb^®^ seems reasonable, but is not sufficient on its own: first, even though vancomycin TDM is widely practiced, the results of the TDM are not immediately available, especially during the weekend [22, 37, 38]. Second, the TDM only provides information at the time of blood collection (but not beyond), i.e., the impact on the relevant PK/PD index AUC is hardly assessable. Third and most important, it can only be assessed a posteriori whether the dosage was adequate, but no dosing recommendation is provided a priori by TDM. On the other hand, our study now provides a clear dosing recommendation a priori. Therefore, we believe that our study considerably contributes to the optimization of vancomycin therapy and subsequently to the successful therapy of severe infections.

Several limitations of our study should be mentioned. First, the number of patients in our study was small, however the estimated parameters could be precisely estimated. In addition, a substantial and clinically relevant effect of CytoSorb^®^ on vancomycin exposure could be demonstrated. Nonetheless, larger studies addressing the adsorption of vancomycin by the CytoSorb^®^ with a denser sampling scheme in the best case with pre- and post-CytoSorb^®^ filter samples are desirable. Previous suggestions indicate an influence of blood contents on adsorption properties (blood of critically ill patients contains variable contents of endogenous compounds and drugs possibly saturating adsorption capacity of the CytoSorb^®^) [17, 26]. However, our study design did not allow to reliably explore interindividual variability in adsorption properties. Secondly, we analyzed the adsorption of vancomycin only in critically ill patients with sepsis or septic shock, although the CytoSorb^®^ can be used in other settings as well. The results of our study are not straightforwardly transferable to other settings and should be reviewed in the future. Finally, we would like to emphasize that our findings do not translate to other drugs and antibiotics and therefore highlight that every drug needs to be investigated separately. The proposed adsorption models might be helpful for future investigations.

## Conclusion

The use of CytoSorb^®^ leads to a clinically significant adsorption of vancomycin (max. 572 mg) in critically ill patients with sepsis or septic shock. We recommend the administration of an additional dose of 500 mg vancomycin over 2 h to avoid subtherapeutic vancomycin exposure.

## Data Availability

The datasets generated and/or analyzed during the present study are not publicly available, but they are available from the corresponding author on reasonable request.

## Abbreviations

AUC: Area under the concentration time curve
MIC: Minimal inhibitory concentration
PK/PD: Pharmacokinetics/pharmacodynamics
TDM: Therapeutic drug monitoring
CVVHD: Continuous venovenous hemodialysis
CVVHDF: Continuous venovenous hemodiafiltration
IIV: Interindividual variability
OFV: Objective function value
CI: Confidence interval
SOFA: Sequential organ failure Assessment
CRP: C-reactive protein
CRRT: Continuous renal replacement therapy
CVVHD: Continuous venovenous hemodialysis
CVVHDF: Continuous venovenous hemodiafiltration
IL-6: Interleukin 6
CL: Clearance
V: Volume of distribution
COV: Covariate
*k*_max_: Maximum adsorption rate constant
*A*_max_: Maximum drug amount adsorbed at the CytoSorb^®^ filter
CV: Coefficient of variation
